# Association of frizzled-related protein (MFRP) and heat shock protein 70 (HSP70) single nucleotide polymorphisms with primary angle closure in a Han Chinese population: Jiangsu Eye Study

**Published:** 2013-01-28

**Authors:** Haihong Shi, Rongrong Zhu, Nan Hu, Jian Shi, Junfang Zhang, Linjuan Jiang, Hong Jiang, Huaijin Guan

**Affiliations:** Eye Institute, Affiliated Hospital of Nantong University, Nantong, China

## Abstract

**Purpose:**

Primary angle closure (PAC) is the early stage of primary angle closure glaucoma (PACG). It is believed that the formation of PAC is regulated by a tissue remodeling pathway. This study investigated the association between gene variants in extracellular matrix metalloprotease-9 (MMP-9), methylenetetrahydrofolate reductase (MTHFR), frizzled-related protein (MFRP), heat shock protein 70 (HSP70), and PAC.

**Methods:**

The study was part of the Jiangsu Eye Study. The sample consisted of 232 subjects with PAC and 306 controls obtained from a population-based prevalence survey conducted in Funing County in Jiangsu Province, China. The single nucleotide polymorphisms (SNPs) included rs17576 and rs3918249 in MMP-9, rs1801133 in MTHFR, rs3814762 in MFRP, and rs1043618 in HSP70. SNP genotyping was performed with a TaqMan MGB probe using the real-time PCR system.

**Results:**

Among the five SNPs tested, only MFRP rs3814762 and HSP70 rs1043618 showed a nominal association with PAC. The frequency of the minor T allele of MFRP rs3814762 was higher in the control group than in the PAC group (uncorrected p=0.016 and p=0.027, for alleles and genotypes, respectively) and conferred an odds ratio (OR) of 0.67 in the allelic analysis, indicating a protective role of the SNP in developing PAC. In contrast, the frequency of the CC genotype of HSP70 rs1043618 was higher in the PAC group than in the control group (uncorrected p=0.048 and p=0.022 for the genotypes general model and recessive model, respectively) and conferred an OR of 1.79 in the recessive model, indicating a harmful role in developing PAC. However, the differences did not remain statistically significant after Bonferroni correction. The remaining three SNPs showed no differences in the distribution of the genotypes and allele frequencies between the two groups.

**Conclusions:**

Our study reveals a suggestive association of MFRP and HSP70 with PAC in a Han Chinese population. The results from this population-based survey will serve as the baseline for prospective observation of the role of tissue remodeling pathway in the development of PACG.

## Introduction

Glaucoma is the second leading cause of irreversible blindness worldwide. Primary glaucoma is classified as primary open-angle glaucoma (POAG) and primary angle closure glaucoma (PACG), depending on the anatomy of the anterior chamber angle. PACG affects as many as 4.5 million people in China, and it has been reported that Asian populations are at higher risk of developing PACG than other ethnic groups [1].

Eyes with PACG display characteristic anatomic features such as a shorter corneal diameter, a steeper corneal curvature, a shallower anterior chamber, a thicker and more anteriorly positioned lens, and a shortened eyeball, often accompanied by hyperopic refraction error [2]. The risk factors for developing PACG include age, female gender, and family history [3]. First-degree relatives were found to have a six- to ninefold increased risk of developing PACG [4]. Siblings of Chinese patients with PAC or PACG have almost a 50% probability of having narrow angles and are more than 7 times more likely to have narrow angles than the general population [5]. Ethnic differences are also associated with PACG. There is a higher prevalence among Inuits and Asians compared to Caucasians, suggesting a genetic predisposition to the disorder [6]. Primary angle closure (PAC) refers to an eye with an occludable drainage angle and features indicating that trabecular obstruction by the peripheral iris has occurred. A diagnosis of PAC includes asymptomatic individuals with occludable angles who have not had an acute attack and those who have had an attack that was treated promptly but suffered no detectable nerve damage. Namely, PAC is the earlier stage of PACG, and the individuals share the same anatomic features mentioned above. Because the ocular anatomic features are the predisposing factors for PACG, genes relating to regulating axial length and structural remodeling of connective tissues may contribute to development of PACG.

Some tissue remodeling genes, including frizzled-related protein (MFRP) [7,8], extracellular matrix metalloprotease-9 (MMP-9) [9-11], and methylenetetrahydrofolate reductase (MTHFR) [12], have been reported to be associated with PACG. Even though heat shock protein 70 (HSP70) does not regulate tissue remolding directly, the gene regulates the expression of matrix metalloproteinases (MMPs) and is thought to be associated with PACG [13]. However, these findings are controversial and have not been replicated by independent studies.

Because of the obvious anatomic features in PAC, we targeted PAC rather than PACG to evaluate the association of tissue remodeling genes with the phenotype. To the best of our knowledge, this is the first report of PAC single nucleotide polymorphism (SNP) association in a Han Chinese population. Moreover, the results from this population-based survey will serve as the baseline for prospective observation of the role of genetic factors in the development of PACG.

## Methods

### Study participants

The study was part of the Jiangsu Eye Study. The age and gender distribution of PAC in Jiangsu Eye Study is summarized in Appendix 1. The study was conducted according to the Declaration of Helsinki, approved by the Ethics Committee of the Affiliated Hospital of Nantong University, and followed the tenets of the Declaration of Helsinki. Each participant was fully informed of the purpose and procedures involved in the study and signed the Informed Consent Form. The patients’ general demographic information is listed in Table 1. All participants were recruited from a population-based prevalence survey using a cluster random sampling strategy conducted in Funing County in Jiangsu Province, China. Of the 6,032 people screened, 232 people with PAC and 306 controls were enrolled in the study. Subjects with PAC and controls were matched for sex and age, and were ethnically homogenous. The participants were unrelated and self-identified Han Chinese. There was no difference between the control group and the PAC group in gender, age, and systemic disease distribution. We took the published SNP data for MFRP and HSP70 PACG association as the references in power analysis [7,13]. Based on a predefined two-sided alpha of 0.05 and the sample size, there was greater than 85% power to detect a ±5% departure from an allele frequency of 20% [7].

**Table 1:** Demographics of study participants.

Demographic features	Control n (%)	PAC n (%)	P
Female	248 (81.05)	191 (82.33)	0.70
Male	58 (18.95)	41(17.67)	
Mean age (year)±SD	65.08±7.53	64.84±8.59	0.74
Age range	50–85	50–83	
Hypertension	66 (19.64)	46 (19.83)	0.69
Diabetes	24 (7.36)	20 (8.6)	0.76
cardiovascular	10 (3.27)	4 (1.72)	0.41

All study participants were residents of Funing County in Jiangsu Province, China, aged 50 years and above, and underwent a detailed ocular examination that included best-corrected visual acuity (BCVA), anterior segment photography, Goldmann applanation tonometry, fundus examination, optic disc photography, visual field, objective refraction, and subjective refraction. The depth of the peripheral anterior chamber was determined using the Van Herick technique [14]. Subjects with a peripheral chamber depth less than one-third of corneal thickness (CT) were invited for gonioscope, A-scan ultrasonography, and ultrasound biomicroscopy (UBM, SW-3200S, SUOER, China) examinations. UBM examinations were conducted in light and dark conditions and in eight positions. The detailed protocol for gonioscopy and UBM came from Barkana et al. [15].

PAC was defined according to the International Society of Geographical and Epidemiologic Ophthalmology (ISGEO) classification by Foster et al. [16]: 1) Either eye has the presence of an occluded angle (at least 180 degrees of closed angle in which the trabecular meshwork is not visible on gonioscopy or iris apposition to the trabecular meshwork more than 180 degrees on UBM), 2) no signs of secondary angle closure, 3) no signs of glaucomatous optic neuropathy and peripheral visual loss, and 4) no previous ocular surgery or laser therapy. The clinical features of the subjects with PAC are listed in Table 2.

**Table 2:** Clinical features of 232 PAC subjects.

Parameters	Right eye	Left eye	Total eyes
Axial length (mm)	22.17±0.83	22.17±0.82	22.17±0.83
ACD (mm)	2.49±0.29	2.45±0.30	2.47±0.29
Refractive (diopter)	0.53±1.85	0.68±1.87	0.58±1.84
Tonometry (mmHg)	15.18±4.31	15.78±4.46	15.52±4.39

The criteria for enrollment of the control group were the following: 1) peripheral chamber depth more than one-third of corneal thickness, 2) intraocular pressure less than 21 mmHg, 3) normal optic nerve heads with a cup-to-cup ratio of less than 0.5, 4) normal visual field, 5) no family history of glaucoma, 6) no ophthalmic diseases except slight cataract, and 7) refractive error less than three diopters.

### Genotyping

Genomic DNA was extracted from the peripheral blood of each individual using the Qiagen Blood DNA Mini Kit (Qiagen, Valencia, CA), according to the manufacturer’s instructions and stored at −20 °C.

The samples were genotyped with the TaqMan genotyping assay (Applied Biosystems, Foster City, CA) using the Real-time PCR 7500 system (Applied Biosystems). The SNP selection from the four genes was based on similar studies that suggested possible functional alteration and disease association of the selected SNPs. Three of the five selected SNPs are missense variations [7-13,17]. Details of the tested SNPs and TaqMan assays are listed in Table 3. PCR reactions were performed in a total volume of 10 µl containing 1 µl (10 ng) DNA, 5 µl TaqMan Universal Master Mix, 0.20 µl TaqMan SNP Genotyping Assay Mix (40X), and 3.8 µl DNase-free, sterile filtered water. Amplification was performed with an initial denaturation at 95 °C for 5 min, followed by 40 cycles of denaturation at 95 °C for 30 s and annealing at 60 °C for 30 s.

**Table 3:** SNPs being tested and assay information from Applied Biosystems.

Gene	SNP ID	ABI assay ID	SNP type	Chr site
MMP9	rs17576	C___1414756_10	Intron	Chr.20: 44,638,136
MMP9	rs3918249	C__11655953_10	Mis-sense (Q279R)	Chr.20: 44,640,225
MFRP	rs3814762	C__27488402_20	Mis-sense (M136V)	Chr.11: 119,216,504
HSP70	rs1043618	C__11917510_10	5-UTR	Chr.6
MTHER	rs1801133	C___1202883_20	Mis-sense (A222V)	Chr.1: 11,856,378

### Statistical analysis

Statistical analysis was performed with SPSS version 15.0 software (Chicago, IL). Differences in age and gender between the subjects with PAC and controls were assessed using the Student *t* test and a chi-square test, respectively. Hardy–Weinberg equilibrium was tested using a chi-square test. To analyze the association of the five SNPs with the PAC group and the control group, the frequency of the genotypes and alleles was evaluated using a chi-square test. P values<0.05 were considered statistically significant. In addition, logistic regression analysis was performed to calculate the odds ratio (OR) value and 95% confidence interval (95% CI). If a positive association was found in the initial analysis, Bonferroni correction was performed. Three genotype models were presented: the general model (common allele homozygotes coded as 1, heterozygotes as 2, and recessive allele homozygotes as 3); the dominant model (common allele homozygotes coded as 1 and heterozygotes plus minor allele homozygotes as 2); and the recessive model (common allele homozygotes plus heterozygotes as 1 and minor allele homozygotes as 2). Logistic regression was performed to adjust for age and gender.

## Results

The call rates of all SNP genotyping were >98%, and the call accuracies were 100% in 10% randomly selected samples. All five SNPs conformed to Hardy–Weinberg equilibrium in the PAC group and in the control group (p>0.05).

Among the five SNPs tested, only MFRP rs3814762 and HSP70 rs1043618 showed a nominal association with PAC (Table 4, Table 5). The frequency of the minor T allele of rs3814762 was higher in the control group than in the PAC group (uncorrected p=0.027 and p=0.016 for the genotypes and alleles, respectively) and conferred an OR of 0.67 (95% CI: 0.49–0.93) in developing PAC, indicating a protective role (Table 4). In contrast, the frequency of the CC genotype of HSP70 rs1043618 was higher in the PAC group than in the control group (uncorrected p=0.048 and p=0.022 for the genotypes general model and recessive model, respectively) and conferred an OR of 1.79 in the recessive model, indicating a harmful role in developing PAC (Table 5). After controlling for age, gender, hypertension, diabetes, and cardiovascular disease through multivariate analyses, the SNPs remained associated with PAC. However, after Bonferroni correction, the significance was lost. The remaining three SNPs showed no differences in the distribution of genotype and allele frequencies between the two groups.

**Table 4:** Allele Frequency of SNPs in control and PAC subjects.

SNP	Call rate (%)	Allele distribution Minor/Major (%)		P	OR (95% CI)
		Control	PAC		
MMP9 rs17576 (G/A)	99.8	171/441 (27.9)	118/344 (25.5)	0.38	0.88 (0.67–1.16)
MMP9 rs3918249 (C/T)	99.8	176/436 (28.8)	117/345 (25.3)	0.211	0.84 (0.64–1.10)
MTHFR rs1801133 (G/A)	99.8	274/338 (44.8)	194/268(42.0)	0.363	0.89 (0.70–1.14)
MFRP rs3814762 (C/T)	99.8	125/487 (20.4)	68/394 (14.7)	0.016	0.67 (0.49–0.93)
HSP70 rs1043618 (G/C)	100	214/398 (35.0)	176/288 (37.9)	0.317	1.14 (0.88–1.46)

All HWE p values>0.05

**Table 5:** Genotype Frequency of SNPs in control and PAC subjects.

SNP	Genotype distribution N (%)			General	Pc 1/Pc 2	Dominant p/OR (95% CI)	Recessive p/OR (95%CI)
		Control	PAC	P value			
MMP9 rs17576	GG	164(53.6)	133(57.6)	0.656	0.77/0.78	0.36/0.85 (0.60–1.20)	0.74/0.91 (0.50–1.65)
	GA	113(36.9)	78(33.8)				
	AA	29(9.5)	20(8.6)				
MMP9 rs3918249	CC	160(52.3)	133(57.6)	0.465	0.55/0.56	0.22/0.81 (0.57–1.14)	0.53/0.82 (0.45–1.50)
	CT	116(37.9)	79(34.2)				
	TT	30(9.8)	19(8.2)				
MTHFR rs1801133	GG	93(30.4)	81(35.1)	0.514	0.81/0.81	0.25/0.81 (0.56–1.16)	0.80/0.95 (0.61–1.46)
	GA	152(49.7)	106(45.9)				
	AA	61(19.9)	44(19.0)				
MFRP rs3814762	CC	196(64.1)	166(71.9)	0.027	0.01/0.02	0.06/0.70 (0.48–1.01)	0.02/0.26 (0.07–0.89)
	CT	95(31.0)	62(26.8)				
	TT	15(4.9)	3(1.3)				
HSP70 rs1043618	GG	123(40.2)	95(41.0)	0.048	0.03/0.03	0.86/0.97 (0.68–1.37)	0.02/1.79 (1.08–2.97)
	GC	152(49.7)	98(42.2)				
	CC	31(10.1)	39(16.8)				

All p values>0.05 after Bonferroni correction Pc 1: p value corrected for age and gender Pc 2: p value corrected for hypertension, diabetes and cardiovascular disease

A Singapore study reported that the anterior chamber width was significantly smaller in women compared to men [18]. To control for possible inabilities to adequately adjust for gender, we performed a supplementary analysis on female participants only to see whether excluding male participants would fundamentally change the pattern of the SNP association. As shown in Table 6, the patterns and strength of MFRP rs3814762 and HSP70 rs1043618 association with PAC are similar to those with the two genders combined (Table 5).

**Table 6:** Genotype Frequency of MFRP rs3814762 HSP70 rs1043618 in female control and PAC subjects.

SNP	Genotype distribution n (%)			General P value	Dominant p/OR (95% CI)	Recessive p/OR (95%CI)
		Control (n=248)	PAC (n=191)			
MFRP rs3814762	CC	154(62.1)	138(72.6)	0.019	0.02/0.62 (0.41–0.93)	0.04/0.27 (0.08–0.95)
	CT	80(32.2)	49(25.8)			
	TT	14(5.6)	3(1.6)			
HSP70 rs1043618	GG	97(39.1)	74(38.7)	0.047	0.94/1.02 (0.69–1.50)	0.02/1.90 (1.11–3.26)
	GC	124(50.0)	81(42.4)			
	CC	27(10.9)	36(18.8)			

## Discussion

This study, to the best of our knowledge, is the first population-based study to investigate the association of tissue remodeling genes with PAC. The design of a population-based study can minimize the sample selection bias often present in a hospital-based case-control study. Furthermore, our study is the first to discuss the association of rs3814762, rs3918249, rs1801133, and rs1043618 with PAC in a Han Chinese population. The results show that rs3814762 in the MFRP gene and rs1043618 in the HSP70 gene are marginally associated with PAC, despite the loss of significance after Bonferroni correction.

Because PACG is associated with small ocular dimensions, we investigated a putative susceptibility gene for extreme hyperopia and nanophthalmos known as MFRP, even though the results were controversial [19,20]. MFRP is predominantly expressed in the retinal pigment epithelium and ciliary epithelial cells of the eye [21,22]. Seko et al. found retina-scleral signaling and composition during ocular axial regulation, in which the retinal pigment epithelium layer was proposed to transmit these signals [23]. SNP rs3814762 leads to a non-synonymous amino acid change from valine to methionine in MFRP. To date, the functional consequence of MFRP rs3814762, a non-synonymous variation, has not been reported. According to the gene function of MFRP in eye development [24], it is highly possible that the variation causes an abnormality in ocular structure in early age, thus conferring a predisposition to PAC. Aung et al. found that SNPs of the MFRP gene in Chinese Singapore subjects were not associated with PACG or with short axial length eyes [9]. Wang et al. also detected no association in a Taiwanese population [8]. Similar to our finding, however, the frequency of the TT genotype was higher in the control group (9%) than in the PACG group (3%) in Wang’s study. The failure to identify an association may have been due to the small sample size of their study (63 in the PACG group and 66 in the control group). Our sample included than 200 subjects in the PAC and control groups. However, significance did not withstand correction for a strict multiple testing, implying that the potential association is marginal. Our results indicate that the T allele of rs3814762 possibly has a protective effect against PAC, but may not be a direct factor for PACG.

HSP70 is a stress-regulating gene, whose expression is triggered when organisms are exposed to stress, hypoxia, or injury [25]. Under these unfavorable conditions, increased levels of HSP are produced in tissues to protect themselves against stress. The function of HSP is twofold. Underexpression of HSP leads to an inefficient stress response and results in loss of neuroprotective function, while overexpression of HSP activates the autostimulatory response and leads to optic nerve neuropathy [26]. Alternatively, HSP70 might contribute to PACG by affecting the expression of MMPs. HSP activates nuclear factor kappa B and activator protein-1 (AP-1), which leads to the activation of MMP-9 transcription [27]. HSP70 rs1043618 maps to the 5′ untranslated region and causes reduced promoter activity [28]. Wu et al. has demonstrated that the CC but not the GC genotype of rs1043618 downregulates HSP70 expression [29]. Underexpression of HSP may downregulate MMP-9 gene activity, which has been shown to be associated with PACG [11]. Ayub et al. has demonstrated that HSP70 polymorphisms were associated with PACG but not primary open-angle glaucoma [13]. In our present study, the genotype analysis suggests an association between rs1043618 and PAC in recessive mode, despite the difference of allele frequencies failing to reach statistical significance.

MMPs are other candidate genes for shorter axial length, a known risk factor for PACG. MMP-9 is thought to degrade type 4 collagen, an important component of the extracellular matrix [30]. Extracellular matrix remodeling is likely to be an important determinant for the short axial length in relatively small eyes. MMP-9 rs17576 leads to a substitution of arginine by glutamine at position 279 and may downregulate the gene activity in extracellular matrix remodeling during ocular development, thus shortening axial length. In recent studies, rs17576 was found to be associated with susceptibility to PACG in an Australian population [17] and a Taiwanese population [11]. However, two subsequent studies were unable to replicate these findings in Singapore and southern China [9,10]. MMP-9 rs3918249 was also associated with PAC in an Australian population [17]. However, no such replication has been conducted in other populations. In our present study, we did not find any association between rs17576 and rs3918249 in MMP-9 and PAC. The discrepancy between results could be due to the difference in the distribution of SNPs among different ethnic groups or differences in sample size. Additionally, important consideration should be given to the fact that the substrates of MMP-9 are gelatin and type 4 collagen, which are not the predominant collagen fibrils in the sclera [31,32].

Recently, hyperhomocysteinemia has been shown to be involved in the terations of the extracellular matrix and in the structural remodeling of connectives [33,34]. MTHFR rs1801133 leads to reduced enzyme activity and mild hyperhomocysteinemia, induces the expression of collagen and α-actin, and causes extracellular matrix remodeling [11]. Michael et al. found that MTHFR rs1801133 was associated with PACG in patients of Pakistani origin [12]. Hyper-homocysteine may affect the overall anterior segment structure and may be involved in enhancing the attachment of the trabecular meshwork to the iris during development and aging [12]. In our present study, the distribution of genotypes of rs1801133 was similar in the PAC group and in the control group.

Our data do not support a solid association of the investigated SNPs with PAC in a Han Chinese population after strict multiple testing corrections. However, the strength of the association between rs3814762 and rs1043618 with PAC is still noteworthy, and may actually indicate important functional variants. Moreover, the results from this population-based survey will serve as the baseline for prospective observation of the role of genetic factors in the development of PACG.

## Appendix 1. Age and gender distribution of PAC in Jiangsu Eye Study.

To access the data, click or select the words “Appendix 1.” This will initiate the download of a compressed (pdf) archive that contains the file.
